# Comparative Genomics and *stx* Phage Characterization of LEE-Negative Shiga Toxin-Producing *Escherichia coli*

**DOI:** 10.3389/fcimb.2012.00133

**Published:** 2012-11-07

**Authors:** Susan R. Steyert, Jason W. Sahl, Claire M. Fraser, Louise D. Teel, Flemming Scheutz, David A. Rasko

**Affiliations:** ^1^Department of Microbiology and Immunology, University of Maryland School of Medicine, Institute for Genome SciencesBaltimore, MD, USA; ^2^Translational Genomics Research InstituteFlagstaff, AZ, USA; ^3^Department of Microbiology and Immunology, Uniformed Services University of the Health SciencesBethesda, MD, USA; ^4^WHO Collaborating Centre for Reference and Research on Escherichia and Klebsiella, Statens Serum InstitutCopenhagen S, Denmark

**Keywords:** *Escherichia coli*, microbial genomics, Shiga toxin, evolution, phage

## Abstract

Infection by *Escherichia coli* and *Shigella* species are among the leading causes of death due to diarrheal disease in the world. Shiga toxin-producing *E. coli* (STEC) that do not encode the locus of enterocyte effacement (LEE-negative STEC) often possess Shiga toxin gene variants and have been isolated from humans and a variety of animal sources. In this study, we compare the genomes of nine LEE-negative STEC harboring various *stx* alleles with four complete reference LEE-positive STEC isolates. Compared to a representative collection of prototype *E. coli* and *Shigella* isolates representing each of the pathotypes, the whole genome phylogeny demonstrated that these isolates are diverse. Whole genome comparative analysis of the 13 genomes revealed that in addition to the absence of the LEE pathogenicity island, phage-encoded genes including non-LEE encoded effectors, were absent from all nine LEE-negative STEC genomes. Several plasmid-encoded virulence factors reportedly identified in LEE-negative STEC isolates were identified in only a subset of the nine LEE-negative isolates further confirming the diversity of this group. In combination with whole genome analysis, we characterized the lambdoid phages harboring the various *stx* alleles and determined their genomic insertion sites. Although the integrase gene sequence corresponded with genomic location, it was not correlated with *stx* variant, further highlighting the mosaic nature of these phages. The transcription of these phages in different genomic backgrounds was examined. Expression of the Shiga toxin genes, *stx_1_* and/or *stx_2_*, as well as the *Q* genes, were examined with quantitative reverse transcriptase polymerase chain reaction assays. A wide range of basal and induced toxin induction was observed. Overall, this is a first significant foray into the genome space of this unexplored group of emerging and divergent pathogens.

## Introduction

Shiga toxin-producing *Escherichia coli* (STEC) isolates can colonize the intestinal tract in animals and humans, and in humans are associated with diarrheal symptoms ranging from mild diarrhea to severe hemorrhagic colitis (Kaper et al., 2004; Manning et al., 2008). Hemolytic uremic syndrome (HUS), although arising in only a minority of colonized individuals, is a serious and sometimes fatal complication resulting from elaboration of the Shiga toxins (Stx; Karch et al., 1999; Kaper et al., 2004). Many STEC disease outbreaks have been caused by a subset of STEC isolates, *L*ocus of *E*nterocyte *E*ffacement (LEE)-positive STEC, that harbor the LEE pathogenicity island and one or more *stx* genes (Yoon and Hovde, 2008). These isolates have often been designated enterohemorrhagic *E. coli* (EHEC), but the current study will use a genomic designation of LEE-positive STEC. The genes carried in the LEE pathogenicity island encode a type III secretion system that transports effector molecules into the host cells (Kaper et al., 2004). LEE-positive O157:H7 has been responsible for the majority of STEC disease outbreaks in the United States; however, non-O157 LEE-positive STEC serogroups are prevalent in other countries and are increasingly found associated with outbreaks in the United States (Brooks et al., 2005; Johnson et al., 2006; Gould et al., 2009). Although the LEE pathogenicity island is known to be an important virulence factor, LEE-negative STEC isolates from diverse serogroups have been found to cause the same severe diarrheal symptoms and HUS (Johnson et al., 2006; Mellmann et al., 2008; Newton et al., 2009; Kappeli et al., 2011). With the exception of the recent O104:H4 outbreak that occurred in Germany (Rasko et al., 2011), non-O157 STEC isolates have received much less scrutiny at the whole genome level than their LEE-positive counterparts.

Shiga toxin, the crucial virulence factor attributed to the progression of HUS, can be identified in two major antigenic forms, Stx1 and Stx2, with Stx2 identified as the more potent form (Boerlin et al., 1999; Friedrich et al., 2002). However, *stx*_1_ and *stx*_2_ allele variants have been identified; LEE-negative STEC, in particular, have been determined to often carry these diverse toxin subtypes (Zhang et al., 2002; Burk et al., 2003; Orth et al., 2007; De Sablet et al., 2008; Slanec et al., 2009). Scant information exists on the potency of the different allelic forms, but one report concluded that both *in vitro* and *in vivo* potencies of Stx2a and Stx2d were greater than Stx2b and Stx2c (Fuller et al., 2011). In addition to the potency of the particular encoded Stx, the amount of Stx produced is thought to play a role in virulence (De Sablet et al., 2008; Neupane et al., 2011). Stx genes are encoded by lambdoid bacteriophages and enhanced levels of *stx* expression has been observed for some isolates in prophage inducing conditions (Zhang et al., 2000; Ritchie et al., 2003). Considerable heterogeneity in both basal and induced levels of *stx*_2_ expression has been reported among LEE-positive O157:H7 isolates (Ritchie et al., 2003; De Sablet et al., 2008; Zhang et al., 2010; Neupane et al., 2011). In comparison, less information is available regarding levels of *stx* expression for LEE-negative STEC isolates.

Qualitatively, lambdoid bacteriophages are composed of non-homologous DNA segments, or modules, that have been exchanged between various prophages, leading to broad genetic diversity even within single isolates (Johansen et al., 2001; Brussow et al., 2004; Casjens, 2005). For example, substantial phage sequence diversity has been noted among the 11 lambdoid prophages within the genome of the LEE-positive O157:H7 Sakai isolate (Brussow et al., 2004), and other LEE-positive O157:H7 isolates (Johansen et al., 2001; Ogura et al., 2006). Although sequence divergence of *stx*-encoding phages has been identified, the gene structure of the *stx* cassettes is less well known, and has been determined for only a few LEE-negative STEC isolates. Along with the assortment of mosaic structures, a variety of chromosomal insertion locations have been identified for *stx*-encoding phages in LEE-positive STEC isolates. These insertion sites include *wrbA*, *yecE*, *torS/T*, *sbcB*, *yehV*, *argW*, *ssrA*, and *prfC* (Ogura et al., 2007). Interestingly, the insertion sites of the *stx* phages in the genomes of the majority of LEE-negative STEC isolates are often different than those determined for LEE-positive STEC isolates, and remain largely unidentified (Garcia-Aljaro et al., 2006, 2009; Prager et al., 2011).

Although production of Shiga toxin is essential for the progression of infection to HUS, STEC utilize many other virulence mechanisms during colonization of the human intestine (Yoon and Hovde, 2008). The tight adherence of the bacterial cell to the colonic epithelium resulting from expression of the *eae* encoded Intimin and Tir proteins encoded by the LEE pathogenicity island is considered an important step in infection. The LEE-positive STEC also utilize other chromosomally encoded adhesins and typically express multiple fimbriae (Toma et al., 2004; Farfan and Torres, 2011). LEE-positive STEC genomes also carry genes encoding autotransporter (AT) proteins that have been associated with virulence (Wells et al., 2010). Many AT proteins expressed by pathogenic *E. coli* have been characterized and determined to either to function as proteases, adhesins, hemagglutinins, or to promote autoaggregation or biofilm formation (Wells et al., 2010). LEE-negative STEC isolates must utilize factors other than the Intimin/Tir complex to adhere, thus the question arises as to whether they only make use of factors already identified in LEE-positive STEC genomes or also use as yet undiscovered chromosomally encoded adherence factors. The long polar fimbrial gene cluster, designated *lpf*_O113_, was identified in the LEE-negative STEC O113:H21 isolate EH41 (Doughty et al., 2002), and subsequently identified in other LEE-negative STEC isolates, as well as some non-O157 LEE-positive STEC isolates (Doughty et al., 2002; Toma et al., 2004). Along with chromosomally encoded virulence factors, pathogenic *E. coli* often harbor a large virulence plasmid encoding a variety of additional virulence factors. Although there is heterogeneity between virulence plasmids carried by a particular *E. coli* pathotype, the plasmids display a greater level of similarity within the pathotype than between pathotypes (Johnson and Nolan, 2009). A single LEE-negative STEC O113:H21 isolate, designated EH41, harbors a virulence plasmid of ∼166 kb, designated pO113 (Newton et al., 2009). Both pO157, commonly carried by O157:H7 isolates, and pO113 carry the *ehxA* gene encoding enterohemolysin and an *espP* gene encoding a serine protease autotransporter of *Enterobacteriaceae* (SPATE; Newton et al., 2009; Ogura et al., 2009). The STEC autoagglutinating adhesion, encoded by *saa*, has been suggested to be unique to LEE-negative STEC isolates (Paton et al., 2001; Toma et al., 2004; Cergole-Novella et al., 2007; Wu et al., 2010) and is encoded on pO113. Additional genes carried on pO113, reported to be unique to LEE-negative STEC, are *epeA*, *sab*, and *subAB* (Paton and Paton, 2005; Cergole-Novella et al., 2007; Herold et al., 2009; Newton et al., 2009; Bugarel et al., 2010; Irino et al., 2010; Wu et al., 2010) encoding, respectively, a SPATE exhibiting protease and mucinase activity (Leyton et al., 2003), an AT family protein contributing to adherence and biofilm formation (Herold et al., 2009) and the subtilase cytotoxin; this virulence factor is an AB_5_ family toxin that displays cytotoxicity in Vero cell assays and is lethal to mice (Paton et al., 2004).

Multilocus sequence typing (MLST) based on housekeeping genes has demonstrated that LEE-negative STEC isolates are evolutionarily divergent (Tarr et al., 2008; Newton et al., 2009; Steyert et al., 2011). Whereas whole genome comparative analysis has been predominately focused on LEE-positive STEC (Ogura et al., 2007, 2009; Eppinger et al., 2011b). The current study focuses on a diverse set of nine LEE-negative STEC carrying various *stx* alleles, and includes a comparison with four complete reference LEE-positive STEC isolates. The genome-wide comparison allowed for identification of genes located outside the LEE pathogenicity island that are shared in the four LEE-positive STEC genomes, but not in the nine LEE-negative STEC, as well as virulence profile comparisons and identification of sequence regions unique to each isolate. Additionally, we characterized the *stx* phages in the LEE-negative STEC isolates in terms of chromosomal insertion site, genetic sequence, and structure, and levels of basal and induced *stx* expression. Insertion sites not previously reported for *stx*-encoding phages were identified. We were also able to demonstrate that in the more highly virulent of the nine isolates examined, despite carrying different *stx* alleles, the phages share similar Q protein sequences and genetic structure directly upstream of the *stxAB* genes.

## Materials and Methods

### Bacterial isolates and growth conditions

Nine LEE-negative STEC isolates were examined in this study; the isolate names, serotypes, and origins are listed in Table 1. These particular isolates were chosen to represent LEE-negative STEC with diverse serotypes and *stx* allele variants as part of a Genomic Sequencing Center for Infectious Diseases (GSCID) project^1^. Bacteria were cultured in Luria–Bertani (LB) broth at 37°C.

**Table 1:** **Characteristics of LEE-negative STEC isolates sequenced in this study**.

Isolate	Serotype	*stx* variant(s)	Origin	Reference	Accession number
7V	O2:H25	*stx*_2g_	Feces of healthy cattle	Leung et al. (2003)	AEXD00000000
94C	O48:H21	*stx*_1a, stx__2a_	Patient with HUS	Paton et al. (1995b)	AFDU00000000
B2F1	O91:H21	*stx*_2d1, stx__2d2_	Patient with HUS	Ito et al. (1990)	AFDQ00000000
C165-02	O73:H18	*stx*_2d_	Patient with bloody diarrhea	Persson et al. (2007)	AFDR00000000
DG131	O174:H8	*stx*_1c, stx__2b_	Sheep	Paton et al. (1995a), Koch et al. (2001)	AFDV00000000
EH250	O118:H12	*stx*_2b_	Child with abdominal cramps	Pierard et al. (1998)	AFDW00000000
MHI813	O8:H19	*stx*_1d_	Bovine feces	Burk et al. (2003)	AFDZ00000000
031	O174:H21	*stx*_2b, stx_2c__	Bowel contents of baby with SIDS	Paton et al. (1992), Paton et al. (1993)	AFDY00000000
S1191	O139:H1	*stx*_2e_	Pig with edema disease	Weinstein et al. (1988)	AFEA00000000

### Genomic DNA extraction, sequencing, and assembly

Genomic DNA was isolated from an overnight culture using the Sigma GenElute kit (Sigma-Aldrich) and was sequenced at the University of Maryland School of Medicine, Institute for Genome Sciences, Genome Resource Center^2^. The genome sequence was generated using 3 kb insert paired-end libraries on the 454 Titanium FLX (Roche) and the raw paired-end sequence reads were assembled with Celera v. 6.0 (wgs-assembler.sourceforge.net). The raw sequence reads are available for each genome sequenced in this study^3^.

### Phylogenetic analysis based on whole genome alignment

The sequence data for *E. coli/Shigella* genomes (Table A1 in Appendix) were downloaded from GenBank and combined with sequence data from the nine LEE-negative STEC isolates in this study for a total of 39 genomes. The genome sequences were aligned with Mugsy (Angiuoli and Salzberg, 2011), and the genomic core alignment, which consisted of ∼2.5 Mb, was parsed from the Mugsy output using methods described previously (Sahl et al., 2011). A phylogenetic tree was inferred using FastTree2 (Price et al., 2010) with *E. fergusonii* isolate 35469 as the outgroup.

### Whole genome sequence comparison

The sequences of the nine LEE-negative STEC genomes were compared in detail to four complete reference LEE-positive STEC genomes (Table A1 in Appendix). These reference isolates were LEE-positive O157:H7 EDL933 (Perna et al., 2001), O111:H- str. 11128 (Ogura et al., 2009), O26:H11 str. 11368 (Ogura et al., 2009), and O103:H2 str. 12009 (Ogura et al., 2009). The shared genomic sequence regions between the 13 isolates were identified using Mugsy (Angiuoli and Salzberg, 2011) as defined above. Sequence regions uniquely shared by subsets of the 13 genomes, or by a single genome, were identified from the Mugsy output using scripts from bx-python^4^ combined with custom python scripts. Putative unique regions were then further characterized using BLAST (Altschul et al., 1997) against the entire sequence set to verify uniqueness of the alignments.

### BLAST score ratio analysis

BLAST score ratio analysis of selected virulence factors was performed as previously described (Rasko et al., 2005). BLAST score ratio (BSR) analysis identifies the level of relatedness between peptide sequences by dividing the protein query BLAST score by the reference BLAST score. The normalized BSR values were visualized using the MultiExperiment Viewer (Saeed et al., 2003).

### PCR screens for genes of interest

Genomic DNA from two collections of *E. coli* isolates was screened by PCR for the presence of genes of interest. These collections consisted of 73 isolates from the environmental *E. coli* ECOR set (all *stx-*negative, Ochman and Selander, 1984) and the diarrheagenic DECA set containing 79 isolates^5^. The gDNA was interrogated for the genes *saa*, *perC1*, and a gene coding for a hypothetical protein (ECO103_2361 from O103:H2 isolate 12009) using primer pairs saa1, perC1, and hyp, respectively (Table 2). These primers were designed to anneal to conserved regions of the genes after examining MUSCLE alignments for regions with no polymorphism. In addition, the LEE-negative STEC isolate 87-1714 was included in the PCR screen as a control (Tarr et al., 2008; Newton et al., 2009; Steyert et al., 2011). Each 20 μL reaction included 30 cycles consisting of 95°C for 30 s, 53°C for 30 s, and 72°C for 40 s. The *E. coli* K12 isolate MG1655 was employed as a negative control, and STEC O48:H21 94C and LEE-positive O157:H7 EDL933 were used as positive controls for *saa* and the other two genes, respectively.

**Table 2:** **Oligonucleotide primers used in this study**.

Primer set	Amplicon size (bp)	Forward sequence (5′–3′)	Reverse sequence (5′–3′)
stx1RT	115	ACCACGTTACAGCGTGTTG	ACTGCGTCAGTGAGGTTCC
stx2RT	104	CAACGGTTTCCATGACAACG	TGAAACCAGTGAGTGACGACTG
rpoART	57	GCGCTCATCTTCTTCCGAAT	CGCGGTCGTGGTTATGTG
saa1	548	GGGAAGCAACTTGACATAAGTAAAGC	ACCACCAATTATGCGAGTTTCTCC
perC1	249	AGGACTGTACCGGAGAGCAG	GACGTATTCTGTTCTCCTGTCC
hyp	214	TATCAGAGCGGTAACTAAGCG	TCTTGCCCAGAATGTGGTG
RTQ1	133	CATCTGCCACTAAACCACG	CAGTCTTTTTGATATTCGCAAC
RTQ2	104	GGCTGCTTCAGACAATAGC	CGTCATCATCACACTGAATCC
RTQ3	98	GACTGATCCCCGAAAAAGTA	CAACCAGCAAGTCATGCAG
RTQ4	104	TTGAAGGTCTGCTCAATTACG	GGCAAAATTCACAAGGTAAGG
RTQ5	154	GACATCATCATGGCGACG	TTTTCTGGTACCGGATTGAG
RTQ6a	100	GGTTAATACCGTCGAAGGTG	ATCCACCAGTAGATCATGCTG
RTQ6b	106	GGATTGATCCCGACTAAAGTG	AATAATCTACCAACAAATCGTGC

### Shiga toxin containing phage sequences and insertion sites

The insertion sites of the phages carrying the Shiga toxin genes were bioinformatically determined for each isolate. The *stx* genes were located in the assembled contigs and the adjacent sequence surrounding the *stx* genes was extracted and subjected to coding sequence (CDS) analysis^6^. The phage integrase gene and genes adjacent to the integrase gene were identified using BLASTp where possible. The gene adjacent to the integrase was designated as the phage insertion site. The *stx* phage sequences were compared using Mauve (Darling et al., 2010). In some cases contigs were bioinformatically linked where appropriate to obtain complete phage sequences. Although the integration site was determined for all *stx* phages, the 3′ end of the phage could not be conclusively identified in three cases.

### Integrase, Q, and Shiga toxin gene phylogeny

Phylogenetic analysis was performed on *stx* gene sequences extracted from the LEE-negative STEC genomes and the four reference LEE-positive genomes. *Q* genes carried by the *stx* phages were identified by BLASTp and were aligned with MUSCLE (Edgar, 2004), to the *Q* gene sequence identified in the STEC EDL933 isolate *stx*_1_- and *stx*_2_-encoding phages. Integrase gene sequences were identified from reference genomes and the LEE-negative STEC genomes in this study. Sequence surrounding BLAST alignments was extracted and integration sites of insertion elements were determined as described above in an iterative process to provide the most complete dataset. For analysis of each of the *stx*, *Q*, and integrase gene phylogenies, the sequences were aligned using MUSCLE (Edgar, 2004) and a phylogeny was inferred with FastTree (Price et al., 2009).

### Mitomycin C phage induction

Overnight cultures of each STEC isolate were diluted 1:500 into fresh LB broth and grown to an OD_600_ of ∼0.35, then divided into separate cultures of equal volume. Mitomycin C at a final concentration of 0.5 μg/mL was added to one of the cultures. The induced and control cultures for each isolate were incubated at 37°C with shaking for 2 h, followed immediately by RNA extraction. The experiment was performed in triplicate for each isolate.

### RNA isolation and quantitative RT-PCR

Total RNA was extracted from 8 mL cultures using the RiboPure Bacteria Kit (Ambion) and treated with DNaseI (Ambion). The RNA concentration was measured using a ND-1000 Spectrophotometer (NanoDrop). SuperScript III Reverse Transcriptase (Invitrogen) with random hexamers was used to prepare cDNA from 1 μg total RNA for each sample. The resulting cDNA, diluted 1:50, was used in quantitative reverse transcriptase polymerase chain reaction (qRT-PCR) reactions performed using Power SYBR Green PCR Master Mix (Applied Biosystems) in a 7900HT Fast Real-Time PCR System (Applied Biosystems). Each 10 μL qRT-PCR reaction contained 2.5 μL cDNA template, 2X SYBR Green mix, and gene specific primers at a concentration of 0.2 μM each. All qRT-PCR reactions were carried out in triplicate for each of the three biological replicates for each condition, and included 40 cycles consisting of 95°C for 15 s followed by 60°C for 1 min. Fluorescence was monitored in a dissociation stage as products were heated from 60 to 95°C to verify primer specificity by melting curve analysis. Transcripts encoding the target genes *stxA*_1_, *stxA*_2_, and *Q*, along with the reference gene, *rpoA*, were detected using primer pairs listed in Table 2. Efficiencies for qPCR reactions were determined using LinRegPCR (Ramakers et al., 2003), and relative expression levels of the target genes in induced versus control cultures for each isolate were calculated from *C*_t_ results and efficiencies using the Pfaffl method (Pfaffl, 2001). Basal level target gene expression for each isolate relative to EDL933 were also calculated from results obtained from control cultures.

Notably, the primers annealing to the A subunit of the Shiga toxin genes were designed to be specific for either *stx_1_* or *stx*_2_; this was verified by examining isolates carrying *stx*_1_ or *stx*_2_ or in combination. The *Q* gene primers were designed to be specific for a particular cluster of *Q* gene sequences as described below; however, there are cases where two *Q* genes with similar sequence are present in a single genome. For example, there is a similar *Q* gene associated with the *stx*_1_ and *stx*_2_ genes in EDL933, thus measured transcript abundance cannot distinguish between *Q* mRNA from the two phages. This is also true for isolates EH250 and 7V.

## Results

### Isolate diversity

**Figure 1:**
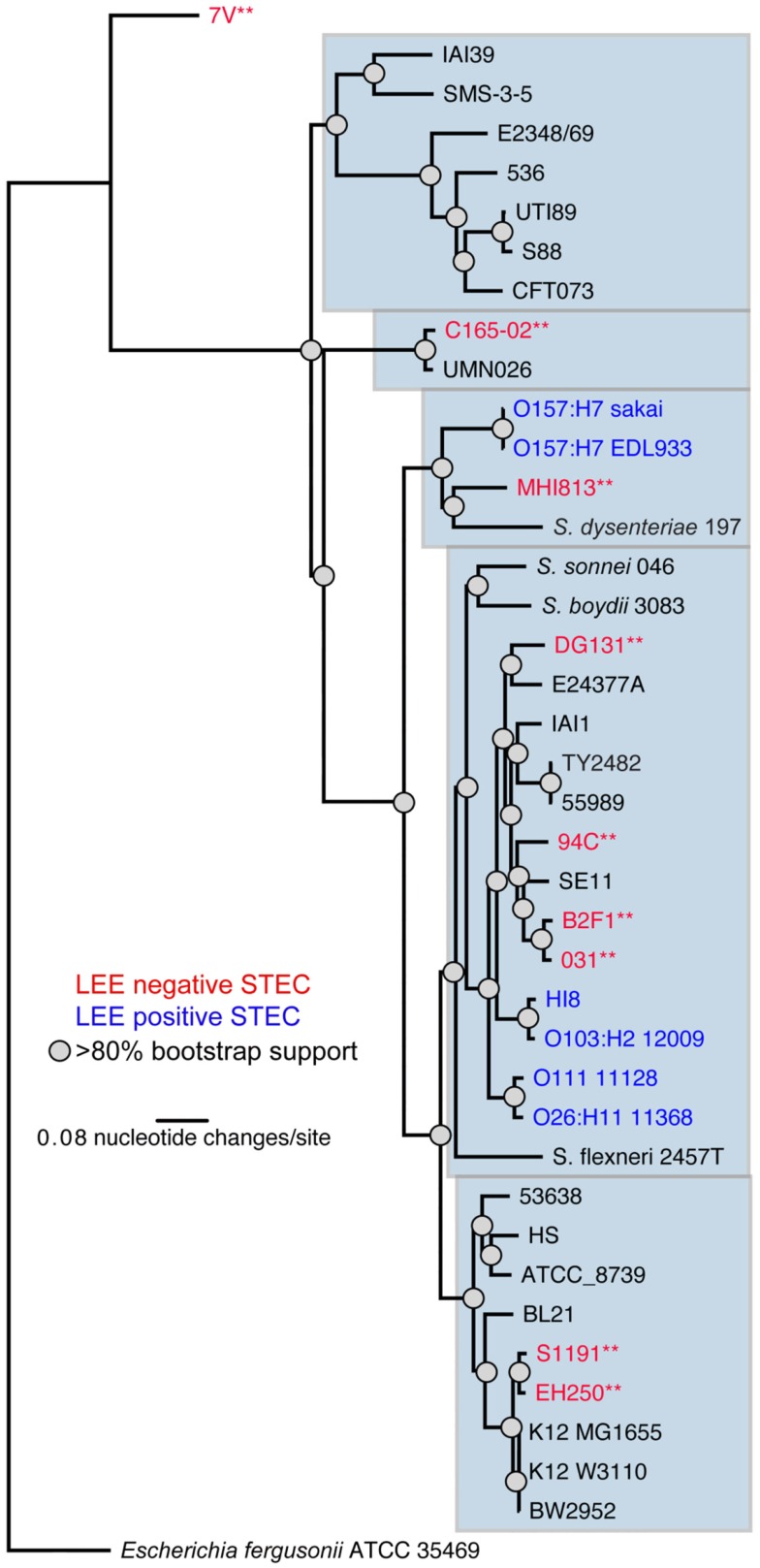
**A whole genome phylogeny of nine LEE-negative (red) and four LEE-positive (blue) STEC compared in this study**. Whole genome sequences for the LEE-negative STEC sequenced in this study (indicated by asterisks) was combined with sequence data obtained from GenBank for *E. coli/Shigella* genomes representing the major pathotypes (Table A1 in Appendix), and aligned based on concatenated regions of shared sequence as determined from analysis using Mugsy (Angiuoli and Salzberg, 2011). The phylogenetic tree was inferred with *E. fergusonii* isolate 35469 as the outgroup.

**Figure A1:**
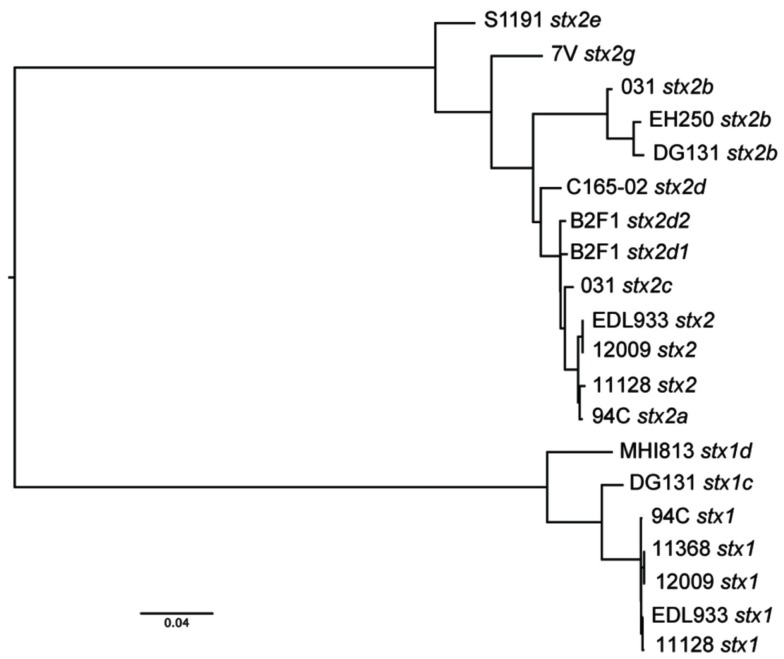
**Shiga toxin gene phylogeny**. A phylogenetic tree was constructed from an alignment of concatenated *stxA* and *stxB* gene subunits for each of the Shiga toxins encoded in the 13 isolates compared in this study.

The nine LEE-negative STEC isolates examined in this study display both whole genome phylogenetic diversity and variation in the Shiga toxin alleles they harbor. A phylogeny was inferred from the conserved genomic core (∼2.5 Mbp) of a diverse set of 39 *E. coli*/*Shigella* genome sequences including representatives of all the major pathotypes (Figure 1). The phylogeny demonstrates that the LEE-negative STEC do not form a tight phylogenetic grouping suggesting that they have evolved multiple times and acquired the *stx* phage multiple times. Additionally, the phylogenetic analysis identified the early evolutionary divergence of the 7V isolate, which had been noted previously by MLST (Tarr et al., 2008; Newton et al., 2009; Walk et al., 2009; Steyert et al., 2011). The MHI813 isolate is more closely related to the EHEC 1 clonal group containing the O157:H7 isolates, while DG131 is the isolate most closely related to the EHEC 2 clonal group. The remaining isolates were distributed throughout the phylogeny. In general, the *stx* gene phylogeny (Figure A1 in Appendix) does not parallel the result found for whole genome phylogenetic analysis. This is not unexpected since *stx* genes are carried on mobile genetic elements.

### Whole genome sequence comparison

Comparative genomics was utilized to determine whether there were any genes shared by all the LEE-negative STEC isolates that were not in the reference LEE-positive STEC genomes, and conversely, whether the LEE pathogenicity island was the only feature that distinguished LEE-positive from LEE-negative STEC. In addition to the nine LEE-negative STEC isolates, four representative LEE-positive STEC genomes were included in the comparative analysis including one from the EHEC 1 clonal group, O157:H7 str. EDL933, two from the EHEC 2 clonal group, O111:H-str. 11128, and O26:H11 str. 11368, and one that is a member of neither group, O103:H2 str. 12009. Whole genome comparative analysis was performed on this set of 13 genomes and identified a shared core alignment length of ∼3.66 Mb. This core sequence size is greater than the ∼2.5 Mb identified when including the 39 isolates used to construct the *E. coli* phylogeny in Figure 1. The whole genome comparison revealed no genomic regions (>500 bp) that are common to all nine LEE-negative STEC and absent in the four LEE-positive STEC genomes. Conversely, in addition to the LEE pathogenicity island, there were six genomic regions identified in all four LEE-positive STEC genomes that were not present in any of the LEE-negative STEC genomes. These include the five non-LEE encoded effectors *espK*, *espN*, *espX7*, *nleA*, and *nleG*, along with two other phage-encoded genes; one gene encodes the transcriptional regulator PerC1 (also termed PchABC in STEC), a homolog of PerC in EPEC, while the other encodes a hypothetical protein (locus tag ECO103_2361 in isolate 12009 and further referred to as *hyp*).

To determine whether the 7V isolate, having diverged earlier from other *E. coli* genomes, was lacking genes that were present in the other 12 genomes. The whole genome comparison revealed that the 7V isolate lacked an 8.9 kb cluster containing seven genes; these genes were identified as Clustered Regularly Interspaced Short Palindromic Repeat (CRISPR)-associated proteins (Barrangou et al., 2007). The reverse analysis (i.e., unique in 7V when compared to the other genomes) identified that 7V contains 120 blocks of sequence >300 bases each, totaling ∼298 kb, that are unique. This quantity of unique sequence was greater than any of the other LEE-negative STEC isolates included in this analysis (Table 3). The number of unique sequence regions >300 bp and total length of unique sequence, along with selected possible virulence factors identified by BLASTX contained in the sequence blocks, are listed in Table 3. Although some putative virulence factors were identified, the majority of the sequence regions contain hypothetical proteins. Other unique regions with predicted functions include phage structural genes and some metabolic-related genes. For example a gene cluster coding for proteins involved in propanediol utilization was discovered in the C165-02 isolate. Overall, the LEE-negative STEC isolates are phylogenetically diverse and each isolate contains features that may contribute to virulence; however further functional analysis will be required to determine the role in virulence, if any.

**Table 3:** **Properties of unique sequence regions and selected factors identified**.

Isolate	#seq*	Total(kb)	Selected factors identified in unique regions
7V	120	298	2 Autotransporters, adhesion/hemaggluntin, type VI secretion Vgr family cluster, DNA transfer protein, 2 major facilitator superfamily transporters, fimbrial protein cluster, F4 fimbriae homolog, fimbrial protein homologs HtrE, PapC, and LpfD, outer membrane protein YopM homolog, reverse transcriptase, serine/threonine phosphatase, RatA-like protein, SWIM zinc finger family protein, tellurite resistance protein TciA, zeta toxin, insecticidal toxin SepC
94C	35	69	2 Adhesin/hemagglutinin, protease regulator PrtR homolog, conjugal transfer proteins including PilT homolog
B2F1	20	35	2 Adhesin/hemagglutinin
C165-02	98	216	Adhesin/invasion TibA homolog, autotransporter adhesion, adhesion/hemagglutinin, AidA-I family autotransporter, type I fimbriae, PapC homolog, type VI secretion family protein, transcriptional regulator YdeO homolog, HtrE homolog, MarR family protein, ArsA, and ArsD, iron uptake IroE and IroN homologs, Clp protease, reverse transcriptase, colicin B, and colicin B immunity proteins
DG131	59	113	3 Hemagglutinin family proteins, type IV secretion pilin homologs PilP, and PilT, FhuA homolog, siderophore receptor IreA homolog, toxin/antitoxin proteins YfjF/YfjZ, reverse transcriptase, colicin E5 immunity protein
EH250	41	91	AfaD homolog, AFA-III adhesion operon regulator, YadA family protein, autoagglutinating adhesion, K88 fimbriae homolog, immunoglobulin binding protein, DprA homolog, capsule polysaccharide biosynthesis family proteins, HipA kinase family protein, SEC-C family protein, MarR homolog
MHI813	86	248	3 Adhesin/hemagglutinins including HecA homolog, AidA-I homolog, 2 fimbrial clusters, type VI secretion system cluster, immunoglobulin A1 protease, AfaC homolog, transcriptional regulator HilD, M23 peptidase family protein, S-type colicin, YkfI/YafW toxin-antitoxin system, RadC, catalase/peroxidase
031	56	163	Adhesion/hemagglutinin, conjugal transfer proteins TraJ and TraX homologs, pilus regulatory protein PapB homolog, fimbrial protein PixA, and PixB homologs, transcriptional regulator YfjR homolog, protein kinase domain protein, ShiA homolog, tellurite resistance protein TehB, reverse transcriptase, programmed cell death toxin MazF
S1191	80	202	Autotransporter EatA homolog, 2 AidA-I autotransporter homologs, hemolysin, type IV secretion conjugal transfer proteins, Kappa-fimbriae cluster, AadA streptomycin resistance, microcin H47

*Number of unique sequence regions >300 bp as determined by Mugsy (Angiuoli and Salzberg, 2011) analysis.

### Virulence profiles of the LEE-negative STEC isolates

Since some of the LEE-negative isolates included in this study cluster more closely with pathotypes other than LEE-positive STEC (Figure 1), we queried the nine LEE-negative STEC genomes using the BSR for virulence factors that are typically associated with pathotypes other than LEE-positive STEC [BfpA (EPEC), AggR (EAEC), PapA (UPEC), STa, STb, LT-A, and LT-B(ETEC); Kaper et al., 2004]. The results revealed the presence of enterotoxin genes typically associated with enterotoxigenic *E. coli* in four of the LEE-negative STEC genomes; the 7V genome encodes a homolog of the heat stable enterotoxin STa, while the S1191 and C165-02 genomes encode STb. The genome of the C165-02 isolate also contains a gene similar to that encoding the B subunit of the heat-labile enterotoxin LT-IIa, while the MHI813 isolate carries a homolog of the gene encoding the A subunit of LT-IIb. These observed intersections of pathotype virulence factors highlight the diversity of *E. coli* as a species as well as the LEE-negative STEC.

**Figure 2:**
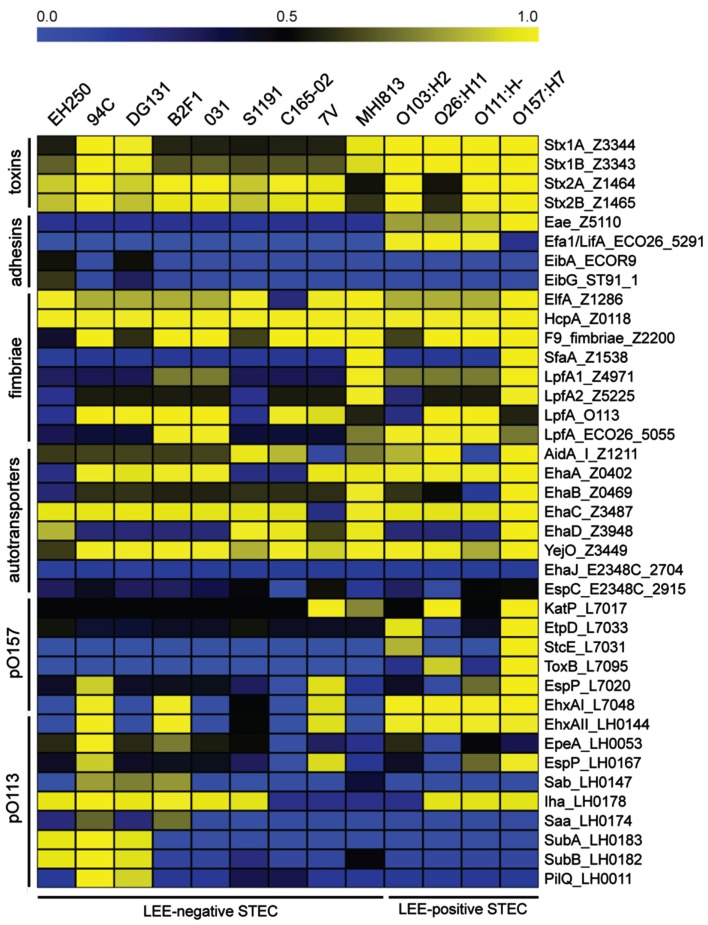
**A virulence gene profile based on BLAST score ratio (BSR) analysis**. BSR analysis was performed on the genomes to determine the presence and level of protein sequence identity of selected virulence factors. Unless an *E. coli* isolate is otherwise indicated in the gene label, reference protein sequences were taken from the LEE-positive O157:H7 EDL933 isolate with the exception of the proteins encoded on pO113, which were taken from STEC O113:H21 isolate EH41. Yellow indicates a higher level of similarity, blue indicates a lower level of similarity, and black indicates ∼50% identity over the length of the sequence queried.

There are few putative virulence factors have been definitively associated exclusively with LEE-negative STEC. Using a BSR analysis we examined the presence and level of sequence similarity of LEE-positive and LEE-negative STEC virulence factors in the 13 genomes (Figure 2). The analysis can be broadly divided into groups of virulence genes: toxins, adhesins, fimbriae, ATs, and plasmid associated genes from pO157 (marker of O157:H7) and pO113 (marker of some LEE-negative STEC; Figure 2). As predicted, all isolates encode one or more of the Shiga toxins (Table 1, Figure 2). The LEE encoded adhesion, intimin, is restricted to the LEE-positive STEC isolates and lacking in the LEE-negative STEC isolates, whereas common fimbriae and ATs are distributed in all types of STEC. The plasmid features appear to be more restricted, but not exclusive, with the LEE-positive STEC isolates containing more pO157 features and the pO113 features being more common among the LEE-negative STEC (Figure 2). Features previously predicted to be restricted to LEE-negative STEC include the adhesin protein Saa, encoded on pO113 (Paton et al., 2001). We confirm that this feature is restricted to the LEE-negative STEC, but is not found widely among LEE-negative STEC isolates. In addition to *saa*, the genes *sab*, *epeA*, and *subAB*, have been reportedly observed only in LEE-negative STEC isolates (Paton and Paton, 2005; Cergole-Novella et al., 2007; Herold et al., 2009; Newton et al., 2009; Bugarel et al., 2010; Irino et al., 2010; Wu et al., 2010). As with *saa*, these genes are present in several of the LEE-negative STEC, but not all. As above, these findings further support the diversity of the LEE-negative STEC isolates within *E. coli*.

### Distribution of genes of interest in *E. coli* collections

Since a limited number of genomes were used in the whole genome analysis, we determined the frequency of the *saa*, *perC1*, and *hyp* genes in a larger collection of *E. coli* genomes. Polymerase chain reaction assays were developed for each of these features, and the prevalence was determined in both the *E. coli* ECOR (environmental) and DECA (diarrheagenic) collections^7^. Among the environmental isolates, only the ECOR37 isolate encodes the LEE pathogenicity island, *perC1* and *hyp*. Seven other ECOR isolates (7/72, 9.7%) also carry *hyp*, but no other isolate contained *perC1*. In the DECA collection, 100% of the EHEC (*LEE*+/*stx*+) genomes (18 of 78 total isolates) harbor both *perC1* and *hyp*, whereas none of the EPEC1 clonal group carries either of the genes. However, the *perC1* gene was found in 100% (24/24), and *hyp* in 58% (14/24) of the remaining LEE-positive *stx*-negative isolates whereas these two genes were present in only 1 of 25 LEE-negative *stx-*negative isolates. The reported absence of the *saa* gene in LEE-positive STEC genomes prompted us to include *saa* in our PCR analysis, which demonstrated the absence of *saa* in all isolates in both the ECOR and DECA collections. These analyses support the previous assertion that Saa is LEE-negative STEC restricted, and that LEE-positive STEC genomes contain *perC1* and *hyp*, but that these genes are not highly conserved among *E. coli* in general.

### Insertion sequence sites and integrase gene phylogeny

**Figure 3:**
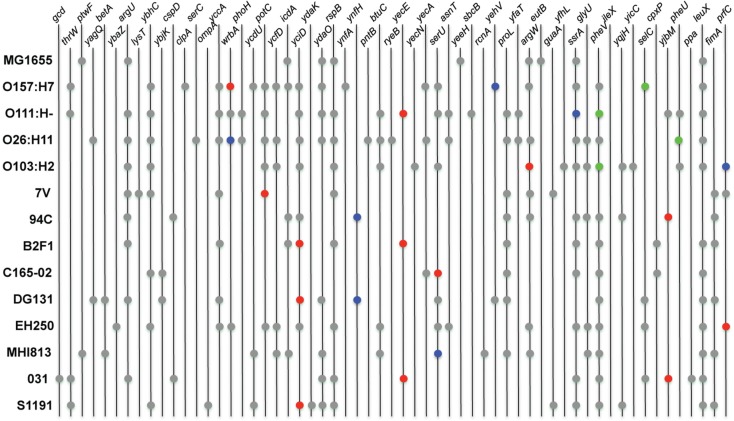
**Chromosomal location of phage integration**. Locations of phage were determined by identifying integrase genes in the genomes of the LEE-negative STEC isolates. Insertion sites were obtained from GenBank for the four reference LEE-positive STEC isolates and *E. coli* MG1655 K12. Prophages encoding *stx_1_* and *stx_2_* are represented in blue and red, respectively. The LEE pathogenicity island is indicated by green, and locations of all other insertion elements are represented in gray.

**Figure A2:**
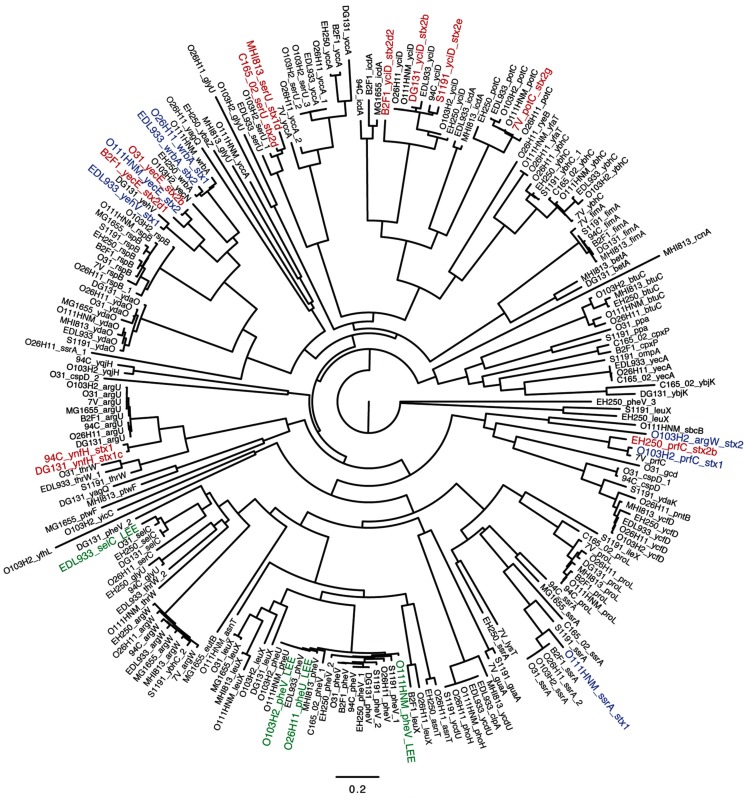
**Relationship between integrase gene phylogeny and chromosomal location of insertion elements**. Integrase gene sequences were extracted from the LEE-negative STEC genomes and the gene adjacent to the integrase gene was designated as the insertion site. Integrase gene sequences were obtained from GenBank for the *E. coli* K12 MG1655 genome along with the four reference LEE-positive STEC genomes. A phylogenetic tree was inferred from an alignment of the integrase genes, and displays the predominant correlation between integrase gene sequence and chromosomal location of the insertion element. Integrase genes extracted from *stx*-encoding phages in the LEE-negative STEC genomes are depicted in red, while those from the reference LEE-positive STEC genomes are depicted in blue and the integrase genes associated with the LEE pathogenicity island are denoted in green. An integrase gene could not be identified in the STEC 94C *stx_2a_* and STEC O31 *stx_2c_* prophages, thus those phages are not included in this analysis.

Several common *stx* phage insertion sites such as *wrbA*, *yecE*, *yehV*, *argW*, *ssrA*, and *prfC* have been reported in LEE-positive STEC genomes (Ogura et al., 2007). Those sites, however, were determined to be unoccupied in many LEE-negative STEC isolates and thus the insertion sites of the *stx* phages in these isolates were essentially unknown (Garcia-Aljaro et al., 2006, 2009; Prager et al., 2011). The *stx* phage insertion sites, as well as the genomic locations of other identifiable phages, were determined in the LEE-negative isolates by examining the integrase genes. Unless a particular insertion site is already occupied, insertion sequences can integrate at preferred locations having a DNA sequence specificity associated with the encoded integrase (Groth and Calos, 2004; Serra-Moreno et al., 2007). The results demonstrate that the *stx* phages are located at a variety of sites in the LEE-negative genomes in this study, many of which appear to be novel insertion sites for *stx* phages (Figure 3). However, this was not because the more widely known insertion sites were already occupied, but rather because of the variety of integrase proteins carried on the phages. The phage integrase sequences were examined, and as expected, integrase phylogeny reveals clusters of genes that utilize the same insertion site (Figure A2 in Appendix). As displayed in Figure A2 in Appendix, there are integrases that are more commonly associated with *stx*-encoding phages; however the integrase sequences are phylogenetically diverse, and no association between a particular *stx* variant and integrase was observed. There are 59 phage insertion sites that have been identified in the 13 genomes examined, but some appear to be more frequently occupied than others (Figure 3). There also does not appear to be an association between phage occupation and phylogeny, as no correlation is seen when the phylogenetic analysis in Figure 1 is combined with the phage insertions sites in Figure 3. This confirms that the phage insertions are governed by the phage integrases and not the core genome, other than containing the insertion site.

### *Stx-*containing phage sequence diversity

**Figure A3:**
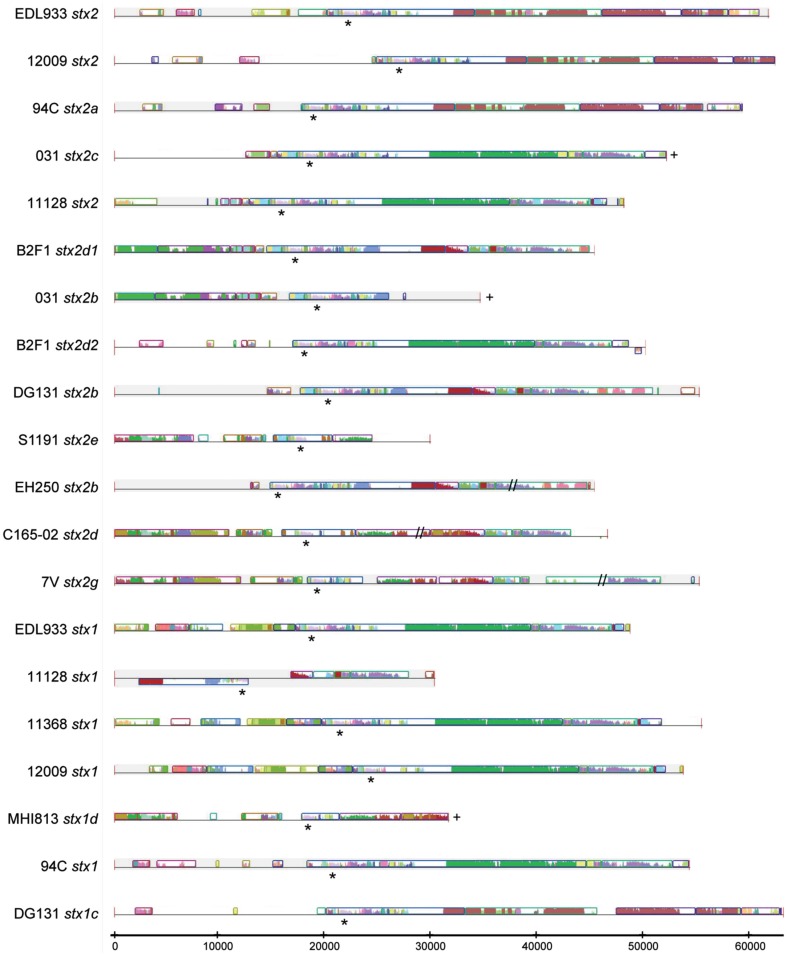
**Sequence comparison of the *stx*-encoding prophages**. Phage sequences extracted from the genomes of the nine LEE-negative STEC isolates and obtained from GenBank for the four reference LEE-positive STEC genomes were subjected to sequence analysis using Mauve (Darling et al., 2010). Similar color denotes regions of shared sequence and the height of the bars denotes level of similarity of the shared sequence regions. Regions where there is a line, but no colored bars, indicate a lack of homology with any of the other phages in the comparison. The location of the *stx* genes is identified with an asterisk (*), the plus (+) signifies that the 3′ end of phage could not be determined unambiguously from the sequence data, and the double hash (//) denotes a gap in known sequence data.

Lambda phages are known to often undergo a significant amount of genetic exchange (Johansen et al., 2001; Brussow et al., 2004; Casjens, 2005). Comparison of the 20 *stx* phage sequences contained in the 13 genomes allowed examination of the potential diversity of the *stx* phages. Complete phage sequences were obtained for the majority of the phages; however, in some draft genomes phage sequences were not contiguous and phages were reconstructed from multiple contigs (Figure A3 in Appendix). In Figure A3 in Appendix, the colored blocks indicate regions of homology and the *stx* genes are indicated by the asterisk. The analysis clearly demonstrates the mosaic nature of the *stx* phages. Furthermore, phages sharing either insertion site or *stx* gene variant often contain extensive non-homologous regions. For example, the B2F1 *stx*_2d2_, DG131 *stx*_2b_, and S1191 *stx*_2e_-encoding phages share the *yciD* insertion site, but display very little sequence homology within the phage. These comparisons suggest a significant degree of diversity among *stx-*containing phages.

### Shiga toxin transcription

**Figure 4:**
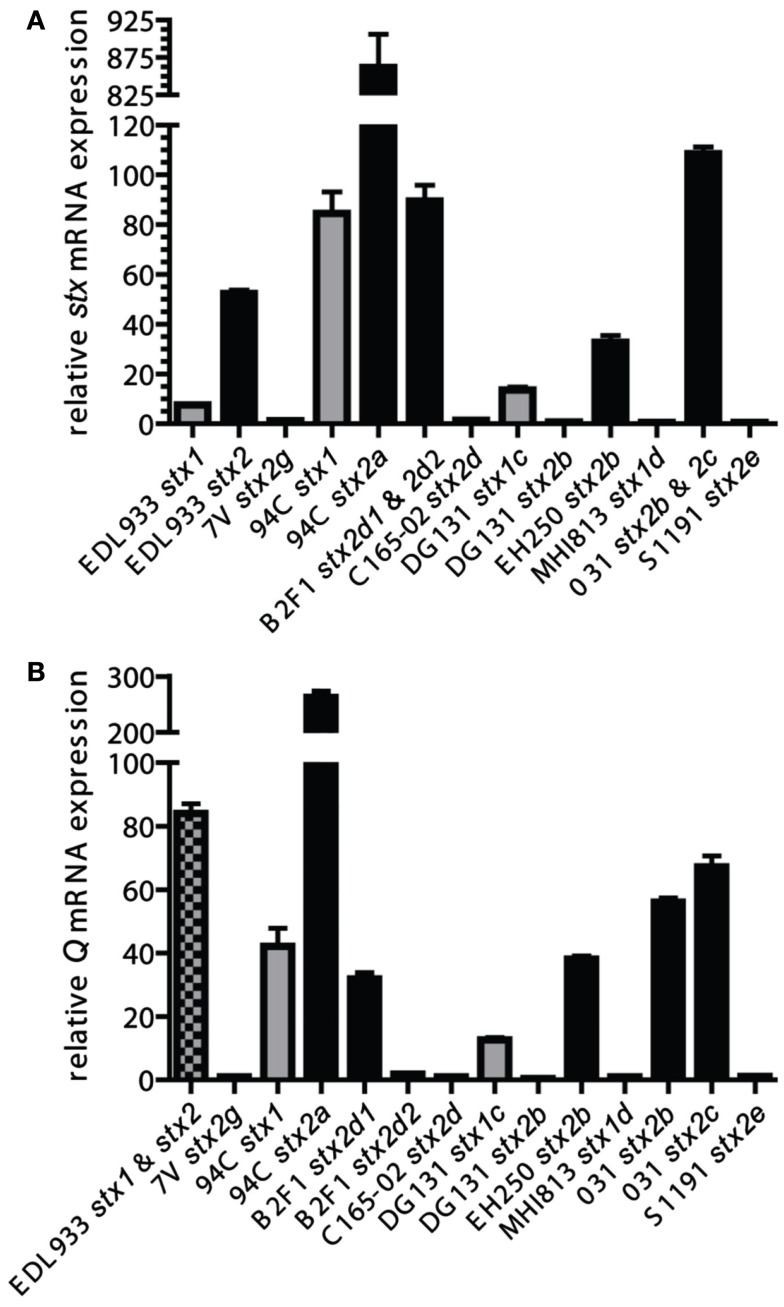
**A comparison of induced *stx* and *Q* gene expression**. Mid-log phase cultures were incubated for 2 h either in the presence or absence of mitomycin C and relative mRNA levels were determined with qRT-PCR. *stx* **(A)** and *Q* **(B)** mRNA expression comparisons were made of mitomycin C-treated cultures relative to un-induced cultures (value of 1 signifies no induction for that particular *stx* in the isolate). Values and standard errors are presented and are based on results from three independent biological replicates each measured with technical triplicates. Results are displayed in gray for *stx_1_*-encoding phages, black for *stx_2_*-encoding phages, and checkered where the expression from the *stx_1_* and *stx_2_* phages could not be distinguished. The *Q* genes associated with the *stx_2b_* and *stx_2g_* phages in isolates EH250 and 7V, respectively, were each found to be associated with another phage in the isolate, thus the measured *Q* expression might have a contribution from that *Q* gene as well.

Potential Shiga toxin induction and production are important as severe complications such as HUS result from the Shiga toxin produced by the bacteria during infection (Karch et al., 1999; Kaper et al., 2004). To determine if the phages in the LEE-negative STEC could be induced to express greater levels of *stx* transcript, mid-log phase cultures were incubated for 2 h either in the presence or absence of mitomycin C, and *stx* gene expression was determined by qRT-PCR. Primers were designed to be specific to either *stx*_1_ or *stx*_2_ alleles; the expression of *stx*_1_ and *stx*_2_ were measured separately in isolates carrying both Shiga toxin types. Two isolates, B2F1 and 031, each harbor 2 distinct *stx*_2_ alleles; however, due to sequence similarity the signal from each *stx*_2_ gene allele could not be determined for these isolates. Levels of *stx* transcripts in induced cultures were normalized to *stx* mRNA levels from untreated cultures for each isolate (Figure 4A). The most highly induced *stx* gene was 94C *stx*_2a_, where the level of induction was over 10 times greater than that observed for EDL933 *stx*_2_. Not only is *stx*_2_ more highly induced in the 94C isolate compared to EDL933, but *stx*_1_ is as well. The results demonstrate that the induction level of the *stx* genes in isolates B2F1 and 031 is also greater than for EDL933 *stx*_2_, but it is not clear if this is due to one of the *stx* genes or both. Elevated levels of *stx* mRNA were not observed under inducing conditions for five isolates. Overall, there does not appear to be a consistent *stx* induction pattern based on STEC genome phylogeny or phage insertion site. Our results also reveal a wide variation in basal level expression of the *stx* genes in the isolates studied. Calculations of the basal and induced expression levels of the *stx*_1_ and *stx*_2_ alleles carried in the LEE-negative STEC isolates relative to those carried by EDL933 are reported in Table A2 in Appendix. From these results it becomes evident that the *stx*_2_ genes are expressed at similar levels in the 94C and EDL933 isolates when induced.

### Q antiterminator phylogeny and transcription

**Figure 5:**
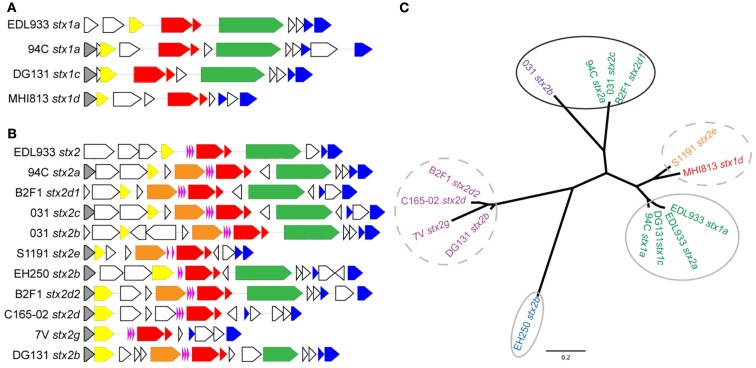
**Gene organization flanking the *stx* genes and *Q* gene phylogeny for the *stx* phages in the LEE-negative STEC isolates and LEE-positive O157:H7 EDL933**. Gene organization comparisons are shown for **(A)** *stx_1_*-encoding phages and **(B)** *stx_2_*-encoding phages. The colors correspond to the following gene designations: gray, *rusA*; yellow, *Q*; orange, DNA methylase; pink, tRNA genes; red, *stxAB*; green, *yjhS*; blue, lysis S, and endolysin genes; white, all other genes, predominantly encoding hypothetical proteins. A cluster diagram based on the *Q* gene sequences was determined **(C)** and primers (Table 2) were designed to be specific for each cluster according to the colors: Q1 green, Q2 purple, Q3 turquoise, Q4 blue, Q5 magenta, Q6a orange, and Q6b red. Clusters circled by a solid black line denote a high level of *stx* induction, gray circles denote intermediate level induction, and broken lines denote lack of induction.

Expression of *stx* genes within lambdoid phages is believed to be largely under the control of the Q antiterminator protein (Brussow et al., 2004). In lambdoid phages the *Q* gene transcription is increased under inducing conditions allowing for increased transcription of the *stx* genes that are downstream of the Q binding site (Brussow et al., 2004). The variety of genetic structures within the Shiga toxin cassettes in the phages can be observed when examining the genes upstream of the *Q* gene through the endolysin gene for each *stx*-encoding phage (Figures 5A,B for *stx*_1_ and *stx*_2_-encoding phages, respectively). Interestingly, the phage gene organization in the vicinity of the Shiga toxin genes 94C *stx*_2a_, B2F1 *stx*_2d1_, and 031 *stx*_2c_ is quite similar and these three phages display the greatest *stx* expression induction. However the genetic architecture does not appear to be the only factor affecting *stx* expression. To further examine the involvement of the Q protein in the regulation of *stx*, the *Q* gene sequences associated with each *stx*-encoding phage were aligned and an inferred phylogeny based on the alignment confirms the broad phylogenetic diversity observed with the whole genome phylogeny (Figure 5C). Interestingly, the three isolates exhibiting the highest level of *stx* induction share similar Q proteins (94C *stx*_2a_, 031 *stx*_2c_, and B2F1 *stx*_2d1_). This suggests that the primary sequence of Q may play a role in the regulation of Shiga toxin, however further experimental evidence is required.

To determine if the induction of the *Q* genes with mitomycin C correlates with the *stx* gene expression, specific primers were designed for each cluster of *Q* gene sequences in an attempt to maximize qRT-PCR efficiency and minimize potential signal from *Q* genes associated with phages in the genome other than the specific *stx*-encoding phage. In the isolates EDL933, EH250, and 7V, the contribution to the *Q* gene qRT-PCR signal from two phages (both *stx-*encoding in EDL933) cannot be distinguished, but independent determination of *Q* expression in the *stx*-encoding phages was possible for all other isolates. The induction pattern for *Q* gene expression parallels the *stx* gene expression, but there is not a perfect quantitative correlation (Figure 4B), suggesting other factors may be involved. These studies confirm that the *stx*_2d1_ gene expression is inducible in isolate B2F1, but not *stx*_2d2_ gene expression (Teel et al., 2002). Our results also indicate that basal level *stx* and *Q* gene expression are not correlated (data not shown), thus expression of *stx* is at least partially dependent on some factor other than levels of *Q* transcripts produced under non-inducing conditions.

## Discussion

Recently, there has been an increased interest in characterizing LEE-negative STEC isolates because certain isolates have been associated with diarrheal symptoms and HUS, as results from infection with certain LEE-positive STEC isolates (Johnson et al., 2006; Mellmann et al., 2008; Newton et al., 2009; Kappeli et al., 2011). Detailed characterization of LEE-negative STEC has indicated that the association to HUS is especially significant for the activatable *stx*_2d_ subtype (Bielaszewska et al., 2006) and that other toxin subtypes are primarily associated with a milder course of disease (Friedrich et al., 2002; Persson et al., 2007). A limited number of reports have partially characterized these *stx*-encoding phages and detailed PCR screens for virulence factors associated with LEE-negative STEC isolates (Muniesa et al., 2000; Recktenwald and Schmidt, 2002; Teel et al., 2002; Cergole-Novella et al., 2007; Beutin et al., 2008; Newton et al., 2009; Wu et al., 2010; Prager et al., 2011), but there remains a paucity of whole genome studies. To fill this knowledge gap a comparative genomics study of nine phylogenetically diverse LEE-negative STEC isolates and four reference LEE-positive STEC isolates was undertaken. Utilizing a gene-independent whole genome alignment method we determined that as a subset of STEC, the LEE-negative STEC isolates, do not share any genes in common that are lacking in all the LEE-positive STEC genomes examined. The phylogenetic diversity of the LEE-negative STEC may preclude the identification of a molecular marker that can differentiate the LEE-negative STEC isolates as a group from all other *E. coli* (Figure 1). Traditionally, LEE-positive STEC isolates are defined as STEC that carry the LEE pathogenicity island in their genome. Our results suggest that genes encoded outside the LEE such as the non-LEE encoded effectors *espK*, *espN*, *espX7*, *nleA*, and *nleG*, as well as the *perC1* gene (also termed *pchABC*) and a hypothetical gene marker, *hyp*, may be suitable biomarkers for LEE-positive STEC. Indeed, the presence of *perC1* and *hyp* in an additional 18 LEE-positive STEC genomes examined, and the lack of these genes in a selection of LEE-negative genomes, suggest that these may be reliable LEE-positive STEC biomarkers. Nonetheless, the set of LEE-negative isolates queried will need to be expanded for a more conclusive result.

The definition of a pathotype of *E. coli* based on a single feature, especially one encoded on a mobile element such as the phage-borne Shiga toxin genes, is likely to reveal highly diverse host isolate backgrounds when examined on a genomic scale. The whole genome phylogeny based on conserved core sequence, utilizing approximately half the genome, determined that the majority of the LEE-negative isolates are more similar to other *E. coli* pathotypes than to LEE-positive STEC (Figure 1). The 7V isolate also appears to be on a deep rooting branch of this phylogeny, previously described as a “cryptic lineage” (Walk et al., 2009). Although the 7V isolate is not phylogenetically related to any prototype ETEC isolates, we determined that it does harbor the heat stable enterotoxin gene STa (ST-IA). These results confirm a recent report that identified the genes encoding STa and KatP carried on the 7V plasmid (Prager et al., 2011). The S1191 and C165-02 isolates also appear to have a STEC/ETEC intermediate pathotype based on virulence factors, as their genomes encode both Stx and heat stable enterotoxin b, STb. Additionally, the C165-02 genome encodes the gene for the B subunit of LT-IIa, whereas the gene coding for the A subunit of LT-IIb was found in the MHI813 genome. As these features are usually plasmid-borne, it is possible that these isolates contain a novel virulence plasmid that is different than pO157, pO113, or the 7V plasmid, but since these are draft genomes it also does not preclude chromosomal insertion of these virulence factors. Without more detailed information from sequencing the isolated plasmids, the comparative genomic analyses suggest that there is a variety of substantially different virulence plasmids harbored by LEE-negative STEC isolates that, in some cases, encode enterotoxin genes.

Without the LEE pathogenicity island, LEE-negative STEC must adhere to the intestinal epithelium by means other than the tight binding brought about by the Intimin/Tir complex (Mellies et al., 2007). The focused analysis on the presence/absence of multiple fimbriae and ATs, some of which may function as adhesions, in the 13 genomes examined, identified further variability (Figure 2). While some of the traditional adhesins were identified in the core of the LEE-negative STEC, additional isolate – specific adhesins and fimbrial genes were identified (Table 3). In fact, additional adherence factors were identified in each of the LEE-negative STEC genomes (Table 3). The combined results of the whole genome sequence comparison, virulence factor profiling analysis and the identification of factors encoded in the isolate-specific sequence regions indicate that there is no common adherence factor in all LEE-negative STEC isolates, but rather that each isolate encodes a particular assortment of adherence factors that allows pathogenic success.

In general, analysis of LEE-positive STEC genomes has revealed the presence of a great number of prophages in each genome, some of which contain virulence-associated genes (Schmidt and Hensel, 2004; Asadulghani et al., 2009). The genomic location of insertion elements and phages in the LEE-negative STEC genomes were cataloged (Figure 3). By inspecting the various insertion site occupancies in the genomes, it is clear that while some genomic sites are occupied by phage more frequently, there appears to be no discernable pattern of phage insertion that correlates to the phylogenetic relationship. Most sites occupied by prophages in the nine LEE-negative STEC genomes are also utilized in at least one of the four LEE-positive STEC genomes, but a few novel insertion sites are identified. We determined that insertion elements are predominately inserted at specific genomic locations that can be correlated to the integrase gene carried on the mobile genetic element (Figure A2 in Appendix). Of note the absence of the LEE pathogenicity island in the LEE-negative STEC genomes is not due to lack of availability of the usual insertion sites adjacent to *selC*, *pheV*, or *pheU* (Figure 3). Interestingly, the *pheV* site is occupied in all LEE-negative STEC isolates, except 7V. The *pheU* site is unoccupied in all nine LEE-negative genomes and the *selC* site is occupied in only the DG131, EH250, and 031 genomes (Figure 3). Thus the LEE pathogenicity island could potentially insert in any of these genomes, but has not.

A comparison of *stx* phage sequences demonstrates the modular structure and sequence heterogeneity present even between phages encoding the same *stx* allele variant (Figures 5 and A3 in Appendix). This heterogeneity, especially in the integrase genes has led to the insertion of *stx*-encoding phages at a variety of genomic locations in the LEE-negative STEC isolates, such that an *stx* allele variant cannot necessarily be correlated with a particular genomic location. As a further example of this fact, we determined the integration site of the *stx*_2e_-encoding phage carried in the S1191 isolate to be *yciD* (Figure 3), whereas, *yecE* is the integration site of the *stx*_2e_-encoding phage in the 2771/97 isolate (Recktenwald and Schmidt, 2002). We also determined that the Q protein sequences are divergent in these two *stx*_2e_-encoding phages, and that the phage gene organization is not shared (Recktenwald and Schmidt, 2002; Beutin et al., 2008; Figure 5B). Q proteins with low sequence identity have been noted previously between LEE-positive O157:H7 *stx*_2c-_encoding phages (Eppinger et al., 2011a) and this work demonstrates the same phenomenon in the LEE-negative STEC isolates (Figure 5C). The extent to which dissimilar Q proteins and/or genetic organization upstream of the *stx* genes affects *stx* expression is not known (Brussow et al., 2004). It is of significance that in a detailed analysis of the *Q* gene sequences, the four Q proteins associated with phages that were not induced by mitomycin C, namely, B2F1 *stx*_2d2_, C165-02 *stx*_2d_, 7V *stx*_2g_, and DG131 *stx*_2b_, are more similar (Figure 5C). Likewise, the *Q* sequences corresponding to the most highly induced *stx* transcript cluster together. In fact, there is a general trend between the *Q* gene induction and the associated *stx* gene induction (Figures 5A,B); however further work would be required to elucidate the reason for the lack of increase in *Q* expression under inducing conditions noted for some of the phages included in this work.

Conflicting reports exist as to whether the Shiga toxin genotype or the level of Shiga toxin production can be used as an indicator for severity of clinical symptoms and progression to HUS associated with STEC infection (Friedrich et al., 2002; Bielaszewska et al., 2006; Orth et al., 2007; De Sablet et al., 2008; Neupane et al., 2011). Not all of the LEE-negative STEC isolates included in this work were isolated from humans, thus a complete comparison aimed at associating Shiga toxin characteristics with virulence in humans cannot be made. All of the Shiga toxins carried in the LEE-negative STEC isolates in this work are prophage-encoded and possibly inducible. Cultures of the LEE-negative STEC and LEE-positive EDL933 were incubated either with or without mitomycin C followed by qRT-PCR utilizing primers having either *stxA*_1_ or *stxA*_2_ as a target (Table 2). Heterogeneity in *stx* expression between isolates has been previously reported (Ritchie et al., 2003; Beutin et al., 2008; De Sablet et al., 2008; Zhang et al., 2010). Variation in basal *stx* expression and level of *stx* induction was observed among the LEE-negative isolates in this work, and a number of the *stx* genes did not appear to be inducible under conditions tested (Table A2 in Appendix and Figure 4A). The isolates demonstrating the greatest induction of *stx_2_* are EDL933, 94C, B2F1, and 031. This induction may be related to the severe clinical outcome associated with each isolate (Table 1) and the potential to exacerbate the disease with the administration of antibiotics. Overall the *Q* gene induction matched the trend of the associated *stx* gene, suggesting that there was a phage-based regulation of the toxin.

In conclusion, this study highlights the broad phylogenetic diversity of LEE-negative STEC isolates as well as the *stx*-encoding prophages harbored in their genomes. Our genome-wide comparative results indicate that LEE-negative STEC isolates as a group vary significantly in the assortment of adhesins and other virulence factors they encode. Sequence comparisons of the *stx*-encoding prophages demonstrate the extensive variation in terms of overall mosaic structures, *stx* allele variants, integrase sequence, Q antiterminator homologs and even the gene organization flanking the *stxAB* genes. These results suggests that extensive genetic exchange has taken place between phages and the possibility may arise from continued genetic exchange. Various genomic insertion sites of the *stx*-encoding phages in the LEE-negative STEC isolates were identified, revealing five sites not previously reported to be utilized by *stx*-encoding phages. The qRT-PCR results of the *stx* and *Q* genes determined that *stx* expression levels are increased in isolates in which *Q* expression levels are also increased under inducing conditions. Finally, this study demonstrates that the overall genome content, phage location and combination of potential virulence factors are variable in the LEE-negative STEC, requiring a larger set of isolates and further functional analyses before conclusions about this group can be made.

## Conflict of Interest Statement

The authors declare that the research was conducted in the absence of any commercial or financial relationships that could be construed as a potential conflict of interest.

## Footnotes

^1^http://gscid.igs.umaryland.edu/

^2^http://www.igs.umaryland.edu/

^3^http://gscid.igs.umaryland.edu/wp.php?wp=emerging_diarrheal_pathogens

^4^http://bitbucket.org/james_taylor/bx-python/wiki/Home

^5^http://www.shigatox.net/

^6^http://www.ncbi.nlm.nig.gov

^7^http://www.shigatox.net/

## Appendix

**Table A1:** ***E. coli*/*Shigella* genomes used in whole genome analysis**.

Name	Accession
IAI39	NC_011750.1
SMS-3-5	NC_010498.1
E2348/69	NC_011601.1
536	NC_008253.1
UTI89	NC_007946.1
S88	NC_011742.1
CFT073	NC_004431.1
UMN026	NC_011751.1
Sakai	NC_002695.1
EDL933	NC_002655.2
*S. dysenteriae* 197	NC_007606.1
*S. sonnei* 046	NC_007384.1
*S. boydii* 3083	NC_010658.1
E24377A	NC_009801.1
IAI1	NC_011741.1
TY-2482	AFOG00000000
55989	NC_011748.1
SE11	NC_011415.1|
H.I.8.	AFDY00000000
2009	NC_013353.1
11128	NC_013364.1
1368	NC_013361.1
*S. flexneri* 2457T	NC_004741.1
53638	NZ_AAKB00000000
HS	NC_009800.1
ATCC 8739	NC_010468.1
BL21	NC_012947.1
K12 MG1655	NC_000913.2
K12 W3110	AC_000091.1
BW2952	NC_012759.1

**Table A2:** ***stx* mRNA expression relative to *stx* expression in EHEC O157:H7 EDL933**.

Isolate	Basal expression*	Induced expression*
***stx1***		
EDL933	1.000 ± 0.052	1.000 ± 0.052
94C	0.427 ± 0.014	4.81 ± 0.51
DG131	0.265 ± 0.012	0.475 ± 0.043
MHI813	0.708 ± 0.140	0.0384 ± 0.0047
***stx2***		
EDL933	1.000 ± 0.025	1.000 ± 0.025
7V	8.67 ± 0.35 × 10^-4^	2.58 ± 0.14 × 10^-5^
94C	0.0728 ± 0.0034	1.12 ± 0.05
B2F1	0.0404 ± 0.0031	0.0679 ± 0.0028
C165-02	7.11 ± 0.30 × 10^-3^	2.21 ± 0.09 × 10^-4^
DG131	5.73 ± 0.42 × 10^-3^	8.21 ± 0.37 × 10^-5^
EH250	0.0182 ± 0.0006	0.0121 ± 0.0011
031	0.182 ± 0.005	0.381 ± 0.018
S1191	8.33 ± 0.11 × 10^-4^	1.07 ± 0.05 × 10^-5^

**Values and standard errors are based on results from three independent biological replicates each measured by qRT-PCR in technical triplicates*.
